# Mpox Clade IIb Virus Introduction into Kinshasa, Democratic Republic of the Congo, July 2025

**DOI:** 10.3390/v18010087

**Published:** 2026-01-08

**Authors:** Tony Wawina-Bokalanga, Eddy Kinganda-Lusamaki, Christian Ngandu, Prince Akil-Bandali, Jérémie Kundey-Mafu, Nadege Ngombe, Laurens Liesenborghs, Princesse Paku-Tshambu, Lorenzo Subissi, Pauline-Chloé Muswamba-Kayembe, Samy Tessi-Mvutukulu, Jacques Santini-Mafuta, Gradi Luakanda-Ndelemo, Olga Ntumba-Tshitenge, Mory Keita, Israel Cinkobu-Bualukengu, Emmanuel Lokilo-Lofiko, Fiston Cikaya-Kankolongo, Sikoti Josaphat, Cris Kacita, Adelar Lofungola, Judith Tete-Sitra, Raphael Lumembe-Numbi, Elzedek Mabika-Bope, Adrienne Amuri-Aziza, Daan Jansen, Jean-Claude Makangara-Cigolo, Jeanine Nkakulu, Yap Boum, Ngashi Ngongo, Sofonias Tessema, Nick Loman, Áine O’Toole, Anne W. Rimoin, Pierre Akilimali, Nicole A. Hoff, Jason Kindrachuk, Steve Ahuka-Mundeke, Martine Peeters, Dieudonné Mwamba, Koen Vercauteren, Andrew Rambaut, Jean-Jacques Muyembe-Tamfum, Placide Mbala-Kingebeni

**Affiliations:** 1 Institut National de Recherche Biomédicale (INRB), Kinshasa P.O. Box 1197, Democratic Republic of the Congo; 2 Service de Microbiologie, Département de Biologie Médicale, Cliniques Universitaires de Kinshasa, Université de Kinshasa, Kinshasa P.O. Box 127, Democratic Republic of the Congo; 3 Department of Clinical Sciences, Institute of Tropical Medicine, 2000 Antwerp, Belgiumdjansen@itg.be (D.J.);; 4 TransVIHMI, Institut National de Santé et de Recherche Médicale (INSERM), Institut de Recherche pour le Développement (IRD), Université de Montpellier, 34090 Montpellier, France; martine.peeters@ird.fr; 5 Institut National de Santé Publique (INSP), Kinshasa P.O. Box 3088, Democratic Republic of the Congo; 6 Hôpital Général de Référence de Kinkole, Kinshasa P.O. Box 243, Democratic Republic of the Congo; 7 Department of Microbiology, Immunology and Transplantation, KU Leuven, 3000 Leuven, Belgium; 8 World Health Organization, CH-1211 Geneva, Switzerland; 9 World Health Organization Country Office, Kinshasa P.O. Box 1899, Democratic Republic of the Congo; 10 World Health Organization Regional Office for Africa, Brazzaville P.O. Box 06, Congo; 11 Graduate School of Cellular and Biomedical Sciences, University of Bern, CH-3012 Bern, Switzerland; 12 Africa Centres for Disease Control and Prevention, Addis Ababa P.O. Box 3243, Ethiopia; 13 Institute of Microbiology and Infection, University of Birmingham, Birmingham B15 2TT, UK; 14 Institute of Ecology and Evolution, University of Edinburgh, Edinburgh EH9 3FL, UK; 15 Department of Epidemiology, Jonathan and Karin Fielding School of Public Health, University of California, Los Angeles, CA 90095, USAnhoff84@ucla.edu (N.A.H.); 16 Kinshasa School of Public Health, University of Kinshasa, Kinshasa P.O. Box 11850, Democratic Republic of the Congo; 17 Department of Medical Microbiology & Infectious Diseases, Max Rady College of Medicine, University of Manitoba, Winnipeg, MB R3E 0J9, Canada; 18 South African National Bioinformatics Institute, University of the Western Cape, Bellville 7530, South Africa

**Keywords:** mpox, mpox virus, MPXV, viruses, Clade II, case investigations, genome sequencing, DRC, Democratic Republic of the Congo

## Abstract

Clade I mpox virus (MPXV) is endemic in the Democratic Republic of the Congo (DRC). Recent studies have described the changing epidemiology of mpox in the country, which has been mainly characterized by the emergence of new MPXV lineages, Clade Ib/sh2023 and Ia/sh2024, associated with sustained human-to-human transmission. Both Clade Ib/sh2023 and Ia/sh2024 are co-circulating in Kinshasa, the capital city of the DRC. Here, we report the first two cases of Clade IIb/sh2017 identified in Kinshasa, DRC, imported from West Africa and locally transmitted. Clinical specimens were collected and tested by PCR. We performed whole genome sequencing using a tiled-amplicon sequencing approach with Clade IIb MPXV-specific primers. The phylogenetic tree shows that Kinshasa Clade IIb MPXV is assigned to Clade IIb/sh2017 within the newly designated lineage G.1, as identified in January 2025 in Sierra-Leone.

## 1. Introduction

Mpox is a zoonotic disease caused by two distinct clades of mpox virus (MPXV); Clade I and Clade II, each divided into two clades, a and b [1,2]. Although the virus can be transmitted through zoonotic spillover, in recent years, at least three different lineages have emerged and spread through sustained human-to-human transmission: Clade IIb/sh2017, Clade Ib/sh2023, and Clade Ia/sh2024 [2,3,4].

The Democratic Republic of the Congo (DRC) has traditionally been the epicenter of Clade I MPXV. For several decades, the country has seen an increasing number of outbreaks caused by zoonotic spillover of Clade Ia [5,6]. In 2024, Clade Ib/sh2023 emerged, prompting the World Health Organization to declare mpox for the second time a public health emergency of international concern [7]. Shortly thereafter, Clade Ia/sh2024 was detected in the capital Kinshasa, where both lineages continue to co-circulate [3,8]. Despite efforts to curb the epidemic, the DRC continues to report the highest number of overall suspected and confirmed mpox cases worldwide [9]. Between 1 January and 20 July 2025, the Ministry of Public Health of DRC, through the Institut National de Santé Publique, reported 61,532 suspected cases of mpox (14,351 PCR confirmed) across all 26 provinces.

On the other hand, human cases of MPXV Clade II were first observed in West Africa [10]. Following the emergence of Clade IIb/sh2017 in Nigeria in 2017 [11], the virus spread internationally and caused the global 2022 mpox outbreak that affected more than 100 countries [12,13]. Although global case numbers have since subsided, Sierra Leone is currently experiencing a large Clade IIb/sh2017 outbreak [14]. In contrast, throughout the global epidemic, the DRC had not reported any cases of Clade IIb/sh2017 to date.

Here, we report the first two Clade IIb/sh2017 mpox cases identified in the DRC, imported from West Africa by the index case and locally transmitted to his wife, with phylogenetic linkage to the ongoing outbreak in Sierra Leone.

## 2. Case Investigations

Between 21 and 26 July 2025, a 45–49-year-old man presented himself to the General Referral Hospital of Kinkole (HGRK—Nsele Health Zone) in Kinshasa, DRC with vesicle lesions. The patient self-reported symptom onset two days before the medical examination, with fever and headache, followed by the appearance of vesicle lesions on the face, palms, and penis, as well as inguinal lymphadenopathy. He had self-medicated for the fever with acetaminophen. He reported no contact or exposure to animals during the preceding three weeks, nor any sexual activities or known contact with MPXV-infected individuals. He also reported a prior history of smallpox vaccination and had a smallpox vaccination scar. He had recently traveled from the Ivory Coast to Togo, and finally to DRC, via transit through Jomo Kenyatta International Airport, Kenya.

Suspecting mpox, the physician immediately transferred the patient to isolation at the mpox treatment unit. Blood and vesicle swab specimens were collected for laboratory analysis. The dual HIV/syphilis Combo rapid diagnostic test (SD Biosensor, Suwon-si, Republic of Korea) was negative for both infections. Initial testing with the Xpert Mpox assay on the GeneXpert system (Cepheid, Sunnyvale, CA, USA), which detects Clade II and non-variola Orthopoxviruses, including Clade I MPXV, at HGRK-detected MPXV Clade II in the vesicle swab sample. Subsequently, vesicle and blood specimens were shipped to the Institut National de Recherche Biomédicale (INRB), Kinshasa, for diagnostic confirmation and whole-genome sequencing of MPXV.

As part of routine contact tracing, nine individuals were identified as high-risk contacts, including the patient’s wife and their three children, one uncle, one nephew, one hairdresser, one renter, and one healthcare worker. Because of sexual contact with the index case, a nasopharyngeal swab was collected from the patient’s wife—aged 35–39 years old—on day 2 after her last exposure to the patient. PCR testing performed at HGRK with the Xpert Mpox assay (Cepheid, Sunnyvale, CA, USA) confirmed the presence of Clade II MPXV DNA in the sample. Consequently, she was immediately admitted to the mpox treatment unit and reported having a fever, swelling of the vulva, and itching. She had vesicle lesions on the face, palms, vulva, and trunk. Vesicle and nasopharyngeal swabs were collected and shipped to INRB, Kinshasa. A follow-up among the other contacts for 21 days is still ongoing.

At INRB, vesicle swab samples of both patients were re-tested with the RADI RP022 mpox detection kit (KH Medical, Pyeongtaek-si, Republic of Korea) on the RADIONE system (KH Medical, Pyeongtaek-si, Republic of Korea), a fully automated point-of-care molecular diagnostic device. In addition, real-time PCR was performed in triplicate on each specimen from both patients (vesicle and blood from the index case, and vesicle and nasopharyngeal swab from his wife) using the RADI FAST RV015R mpox detection kit (KH Medical, Republic of Korea), also following the manufacturer’s instructions.

Following PCR confirmation, multiplex tiling PCR was performed using the Clade IIb MPXV primer pools designed by Chen et al. [15]. The library was also prepared in triplicate using the rapid sequencing DNA V14 barcoding kit (SQK-RBK114.96; Oxford Nanopore Technologies (ONT), Oxford, UK), following the manufacturer’s instructions. The sequencing library was loaded on a R10.4.1 flow cell and run on the GridION sequencer.

MPXV consensus genomes were generated by processing concatenated FASTQ files using the artic-mpxv-nf workflow v2.1.0 (https://github.com/artic-network/artic-mpxv-nf, accessed on 27 July 2025), and the Clade II MPXV genome (GenBank ID: NC_063383.1) was used as a reference. The Nextclade online tool (https://clades.nextstrain.org/, accessed on 27 July 2025) was used to assign the clade of MPXV genomes. Multiple sequence alignment against the Clade II MPXV reference genome (GenBank ID: NC_063383.1) and APOBEC3 mutation analysis were performed using SQUIRREL (https://github.com/aineniamh/squirrel, accessed on 27 July 2025). A phylogenetic tree was inferred using IQ-TREE v2.1.4 [16] with the HKY substitution model [17].

PCR results obtained from the RADIONE indicated amplification cycle threshold (Ct) values of 19.09 and 19.03 for Clade II MPXV in vesicle samples from the index case and his wife, respectively. The RADI FAST real-time PCR assay showed mean Ct values for Clade II of 21.47 and 28.62 in the vesicle and blood specimen triplicates from the first patient, and mean Ct of 21.23 and 34.68 in the vesicle and nasopharyngeal specimen triplicates from the patient’s wife (Table 1).

**Figure 1:**
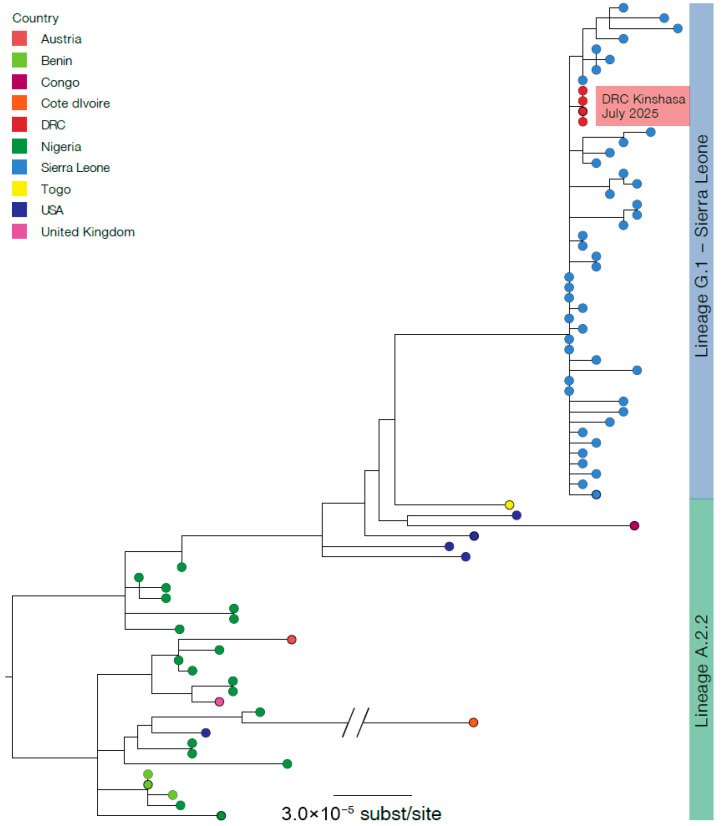
Phylogenetic tree of MPXV sequences from two confirmed cases of Clade IIb/sh2017, Kinshasa, Democratic Republic of the Congo, July 2025 (n = 4 sequences). Sequences are divided into two lineages: Lineage A.2.2 (green) and Lineage G.1 (light blue). Red dots indicate Clade IIb/sh2017 MPXV sequences from this study.

Of the index patient, MPXV genomes were generated from the vesicle swab and blood sample with horizontal genome coverages of 94.19% and 93.39%, respectively. Of the index patient’s wife, the vesicle and nasopharyngeal swabs generated MPXV genomes with horizontal genome coverages of 93.90% and 81.85%, respectively. The four MPXV genomes were similar, assigned to Clade IIb/sh2017 within the newly designated lineage G.1, and clustered with MPXV Clade IIb/sh2017 sequences recently identified in Sierra-Leone (https://virological.org/t/genomic-epidemiology-of-mpox-virus-in-sierra-leone/995, accessed on 5 August 2025) (Figure 1).

Although MPXV Clade IIb/sh2017 sequences obtained from two cases identified in July 2025 in Kinshasa, DRC, are genetically identical and cluster with sequences from the 2025 Sierra Leone mpox outbreak, they differ by two APOBEC3-like mutations (C92702T and C182135T) and one non-APOBEC3 mutation (C161570A), when compared with reference genomes PP_00341CY.3 and PP_00341DW.3 (Pathoplexus accession numbers). In addition, Clade IIb/sh2017 sequences from this study exhibit APOBEC3 mutation enrichment, with approximately 69% (67/97) of reconstructed SNPs consistent with APOBEC3 editing (Table 2). Together, this study indicates that transmission of MPXV Clade IIb/sh2017 in Kinshasa, DRC, resulted from human-to-human transmission.

A recent analysis by Campbell AKO et al. on the genomic epidemiology of Clade IIb circulation in Sierra Leone provided additional insights into the linkages between ongoing circulation in West Africa and the public health impacts of Clade IIb in Sierra Leone in 2025 [18]. This investigation identified linkages between Clade IIb circulation in Sierra Leone with a common ancestor from Nigeria, and linkages to additional regions in West Africa, including Togo. These observations, as well as the continued reporting of infections in multiple West African countries, highlights the ongoing circulation of Clade IIb MPXV more broadly in this region of the continent, the risks for cryptic circulation and ongoing expansion of the virus to new non-endemic regions of the continent, and the need for increased healthcare access and resilience, as well as community engagement regarding mpox.

## 3. Conclusions

The co-circulation of Clade Ib/sh2023 and Clade Ia/sh2024, with the novel importation of Clade IIb/sh2017 and its potential spread in Kinshasa -the largest metropolitan area of the DRC, with international and national connections- warrants enhanced genomic and epidemiological surveillance in the region. This finding underscores the urgent need to improve rapid detection and isolation of mpox cases, and enhance contact-tracing efforts, as well as strengthen genomic surveillance and implement vaccine strategies to mitigate the risk of widespread dissemination of Clade IIb/sh2017 and other MPXV variants in general across DRC provinces.

## Figures and Tables

**Table 1:** Amplification Ct values obtained by PCR RADI FAST with RV015R mpox detection kit and genome coverage of concatenated Clade IIb/sh2017 MPXV genomes, Democratic Republic of the Congo, July 2025 (n = 2 cases).

Patient	Sample ID	Sample Type	Ct Value				Consensus Genome Coverage
			Orthopox	Clade I	Clade II	IPC	
Index case	25MPX-284865V _(a)_	Vesicle	21.18	Neg.	21.64	23.92	94.13%
	25MPX-284865V _(b)_	Vesicle	20.98	Neg.	21.42	23.93	
	25MPX-284865V _(c)_	Vesicle	20.88	Neg.	21.35	23.92	
	25MPX-284865B _(a)_	Blood	29.09	Neg.	28.45	25.14	93.28%
	25MPX-284865B _(b)_	Blood	29.45	Neg.	28.99	25.70	
	25MPX-284865B _(c)_	Blood	29.39	Neg.	28.42	25.84	
Index case’s wife	25MPX-284866V _(a)_	Vesicle	20.29	Neg.	20.58	25.50	93.90%
	25MPX-284866V _(b)_	Vesicle	21.27	Neg.	21.58	26.43	
	25MPX-284866V _(c)_	Vesicle	21.16	Neg.	21.53	26.42	
	25MPX-284866NP _(a)_	Nasopharyngeal	35.54	Neg.	34.67	21.92	81.85%
	25MPX-284866NP _(b)_	Nasopharyngeal	34.13	Neg.	34.63	22.42	
	25MPX-284866NP _(c)_	Nasopharyngeal	35.24	Neg.	34.73	22.55	

Ct: Cycle threshold; ID: Identification; B: Blood; V: Vesicle; NP: Nasopharyngeal; Neg: Negative; IPC: Internal Positive Control; MPXV: mpox virus; Letters a, b, and c in brackets are indicative of technical triplicates.

**Table 2:** Mutation profile within newly generated Clade IIb/sh2017 MPXV genomes, Democratic Republic of the Congo, July 2025 (n = 2 cases).

Mutations	Intergenic	Nonsense	Nonsynonymous	Synonymous	Total
Non-APOBEC3	8	0	12	10	30
APOBEC3-like	7	1	37	22	67
TC → TT	3	1	15	10	
GA → AA	4	0	22	12	

## Data Availability

Genomic sequences recovered in this work have been submitted to Pathoplexus database and have the following accession numbers: PP_0033A47.1; PP_0033A55.1; PP_0033A63.1; and PP_0033A71.1.

## References

[1] World Health Organization (WHO) (2022). Monkeypox: Experts Give Virus Variants New Names. https://www.who.int/news/item/12-08-2022-monkeypox--experts-give-virus-variants-new-names.

[2] Vakaniaki E.H., Kacita C., Kinganda-Lusamaki E., O’Toole A., Wawina-Bokalanga T., Mukadi-Bamuleka D., Amuri-Aziza A., Malyamungu-Bubala N., Mweshi-Kumbana F., Mutimbwa-Mambo L. (2024). Sustained human outbreak of a new MPXV clade I lineage in eastern Democratic Republic of the Congo. Nat. Med..

[3] Wawina-Bokalanga T., Merritt S., Kinganda-Lusamaki E., Jansen D., Halbrook M., O’Toole A., Pukuta-Simbu E., Vakaniaki E.H., Ola-Mpumbe R., Kwete-Mbokama P. (2025). Epidemiology and phylogenomic characterisation of two distinct mpox outbreaks in Kinshasa, DR Congo, involving a new subclade Ia lineage: A retrospective, observational study. Lancet.

[4] Ruis C., Lusamaki E., O’Toole A., Otieno J.R., Colquhoun R., Roemer C., Wawina-Bokalanga T., Tshiani-Mbaya O., Jansen D., Makangara J.C. (2025). A systematic nomenclature for mpox viruses causing outbreaks with sustained human-to-human transmission. Nat. Med..

[5] Bangwen E., Diavita R., De Vos E., Vakaniaki E.H., Nundu S.S., Mutombo A., Mulangu F., Abedi A.A., Malembi E., Kalonji T. (2025). Suspected and confirmed mpox cases in DR Congo: A retrospective analysis of national epidemiological and laboratory surveillance data, 2010–2023. Lancet.

[6] Kinganda-Lusamaki E., Amuri-Aziza A., Fernandez-Nunez N., Makangara-Cigolo J.C., Pratt C., Vakaniaki E.H., Hoff N.A., Luakanda-Ndelemo G., Akil-Bandali P., Nundu S.S. (2025). Clade I mpox virus genomic diversity in the Democratic Republic of the Congo, 2018-2024: Predominance of zoonotic transmission. Cell.

[7] World Health Organization (WHO) (2024). WHO Director-General Declares Mpox Outbreak a Public Health Emergency of International Concern. https://www.who.int/news/item/14-08-2024-who-director-general-declares-mpox-outbreak-a-public-health-emergency-of-international-concern.

[8] Wawina-Bokalanga T., Akil-Bandali P., Kinganda-Lusamaki E., Lokilo E., Jansen D., Amuri-Aziza A., Makangara-Cigolo J.C., Pukuta-Simbu E., Ola-Mpumbe R., Muyembe M. (2024). Co-circulation of monkeypox virus subclades Ia and Ib in Kinshasa Province, Democratic Republic of the Congo, July to August 2024. Eurosurveillance.

[9] World Health Organization (WHO) (2025). Global Mpox Trends. https://worldhealthorg.shinyapps.io/mpx_global/.

[10] Likos A.M., Sammons S.A., Olson V.A., Frace A.M., Li Y., Olsen-Rasmussen M., Davidson W., Galloway R., Khristova M.L., Reynolds M.G. (2005). A tale of two clades: Monkeypox viruses. J. Gen. Virol..

[11] Yinka-Ogunleye A., Aruna O., Ogoina D., Aworabhi N., Eteng W., Badaru S., Mohammed A., Agenyi J., Etebu E.N., Numbere T.W. (2018). Reemergence of Human Monkeypox in Nigeria, 2017. Emerg. Infect. Dis..

[12] World Health Organization Multi-Country Monkeypox Outbreak in Non-Endemic Countries. https://www.who.int/emergencies/disease-outbreak-news/item/2022-DON385.

[13] Wawina-Bokalanga T., Vanmechelen B., Logist A.S., Bloemen M., Laenen L., Bontems S., Hayette M.P., Meex C., Meuris C., Orban C. (2024). A retrospective genomic characterisation of the 2022 mpox outbreak in Belgium, and in vitro assessment of three antiviral compounds. EBioMedicine.

[14] Mitja O., Watson-Jones D., Choi E.M., Jalloh M.B., Sahr F. (2025). Clade IIb mpox outbreak in Sierra Leone. Lancet.

[15] Chen N.F.G., Chaguza C., Gagne L., Doucette M., Smole S., Buzby E., Hall J., Ash S., Harrington R., Cofsky S. (2023). Development of an amplicon-based sequencing approach in response to the global emergence of mpox. PLoS Biol..

[16] Minh B.Q., Schmidt H.A., Chernomor O., Schrempf D., Woodhams M.D., von Haeseler A., Lanfear R. (2020). IQ-TREE 2: New Models and Efficient Methods for Phylogenetic Inference in the Genomic Era. Mol. Biol. Evol..

[17] Hoang D.T., Chernomor O., von Haeseler A., Minh B.Q., Vinh L.S. (2018). UFBoot2: Improving the Ultrafast Bootstrap Approximation. Mol. Biol. Evol..

[18] Campbell A.K.O., Sandi J.D., Omah I.F., Faye M., Parker E., Brock-Fisher T., Gigante C.M., Folorunso V., Kamara M.S., Williams A.J. (2025). Genomic epidemiology uncovers the origin of the mpox epidemic in Sierra Leone. medRxiv.

